# Effects of teacher explicit instruction in stance-taking on students’ perceptions of stance and on their academic writing beliefs

**DOI:** 10.3389/fpsyg.2023.1140050

**Published:** 2023-06-12

**Authors:** Lu Zhang, Lawrence Jun Zhang, Ting Sophia Xu

**Affiliations:** ^1^College of Foreign Languages, Ocean University of China, Qingdao, China; ^2^Faculty of Education and Social Work, University of Auckland, Auckland, New Zealand

**Keywords:** SFL engagement, explicit instruction, perceptions of stance, writing beliefs, EFL students

## Abstract

Scholars have underscored the importance of raising students’ awareness and understanding of stance-taking in academic writing. However, studies on the effects of the pedagogical intervention are just a few. To strengthen this line of inquiry, this paper reports on an intervention study with explicit instruction of stance metalanguage based on the Systemic Functional Linguistics (SFL) Engagement framework and its effects on EFL students’ perceptions of stance as well as on their beliefs about academic writing. A treatment group (*n =* 26) and a comparison group (*n* = 24) were involved. An eight-week writing intervention was provided in the treatment group, while the comparison group received regular curriculum-based instruction. Data from multiple sources were collected prior to and after the writing intervention, including two five-point Likert-scale questionnaires, semi-structured interviews, and reflective journals, to examine possible changes in students’ self-reported perceptions of stance and writing beliefs. Results showed that the intervention was effective in enhancing students’ stance awareness and transactional writing beliefs. Qualitative results further revealed that while the comparison group retained a preference for tentative stance after the writing instruction, intending to avoid potential challenges from readers, the treatment group exhibited a shift in preference for assertive stance valuing the strengths of claims. The treatment group further exhibited an inclination to adopt a wider range of stance options for various rhetorical purposes. Pedagogical suggestions are discussed.

## Introduction

The past decade has witnessed the transformation of academic writing as impersonal discourse into a persuasive endeavor in which a writer uses linguistic resources to convey ideas and address social relations (Aull and Lancaster, 2014; Lee and Deakin, 2016; Zou and Hyland, 2020). In the research on academic writing, authorial stance has recently garnered increasing attention, which refers to writers’ expressions of personal attitudes, evaluations, and interaction with putative readers (Hyland and Sancho Guinda, 2012; Aull and Lancaster, 2020; Zhang and Zhang, 2021a, 2023). Developing a clear and effectual stance is arguably crucial for achieving persuasive argumentation and successful academic writing for both expert and novice writers (Chang, 2016; Crosthwaite and Jiang, 2017; Xu and Zhang, 2019). However, novice writers, especially novice English as a foreign language (EFL) writers, are ineffective in manipulating stance-taking or voice appropriately in writing, and they are also unclear about how to accomplish this goal (Wu, 2007; Zhang, 2013, 2022; Sawaki, 2014; Lee and Deakin, 2016). It has been argued that this situation is attributed to the fact that stance-taking is rarely or tacitly discussed in writing instruction, especially in the context of EFL writing, which obfuscates this valued quality of academic writing to students (Aull and Lancaster, 2020). Stance scholarship thus frequently underscores the importance of facilitating learners’ stance awareness and understanding, and equipping them with a robust metalanguage for consciously monitoring language choices in writing (Chang and Schleppegrell, 2011; Lancaster, 2014; Jou, 2019; Morton and Storch, 2019; Zhang and Zhang, 2021a). Following this strand, a few intervention studies with the explicit teaching of stance metalanguage have been conducted and students have been found to make considerable progress in stance-taking practices in writing (e.g., Chang and Schleppegrell, 2016; Crosthwaite and Jiang, 2017; Jou, 2019; Zhang and Zhang, 2021b). However, it remains unclear if and how such an instruction might affect students’ awareness and understanding of stance, as well as their more general understanding of academic writing. These aspects are influential on how a writer convey ideas, engage readers in the writing process, and take stances in the written product (Graham et al., 1993; Neely, 2014; Chang, 2016). Given the research gap, this study aimed to examine the effects of explicit instruction of stance metalanguage based on the Systemic Functional Linguistics (SFL) Engagement framework (Martin and White, 2005; see also Huang and Zhang, 2022) on EFL students’ understandings of stance and writing, particularly focusing on two psychological factors: learners’ perceptions of stance and their writing beliefs.

## Literature review

### SFL engagement and writing instruction

The SFL Engagement framework as a subsystem of the Appraisal framework (Martin and White, 2005) is informed by the theoretical perspective of dialogism (Bakhtin, 1986). From the dialogic lens, a writer is constantly engaged in a living heteroglossia in the writing process, in which he or she dynamically interacts with referred voices that reflect shared or alien positions in the social and disciplinary community, and at the same time responds to the prospective answers from readers. Based on this, the SFL Engagement framework provides a taxonomy of linguistic resources that writers use to construe intersubjective dialog with alternative views and evaluations, and thus offers a robust metalanguage for analyzing and teaching stances in academic texts. The framework classifies stance-taking utterances in terms of monoglossia (single-voiced) and heteroglossia (multiple-voiced), with heteroglossia further divided into two broad categories (*dialogic contraction and dialogic expansion*) according to the interpersonal functionality of stance resources. Dialogic contraction reduces dialogic possibilities and includes two sub-categories: *disclaim* and *proclaim*. The locutions of disclaim either invoke denial or present countering positions (e.g., *not*, *but*). Proclaim locutions act to limit the scope of dialogistic alternatives through concurrence (e.g., *certainly*), pronouncement (e.g., *indeed*), or endorsement (e.g., the study *shows*…). Dialogic expansion is more open for alternative voices and consists of two sub-categories: *entertain* and *attribute*. Entertain locutions indicate the authorial position as one of many possible positions (e.g., *may*, *possibly*). Attribute locutions indicate authorial acknowledgement or distancing of external sources (e.g., *according to*).

The explicit emphasis of the SFL Engagement framework on evaluative resources in meaning-making has enabled researchers to describe and visualize stances in written discourse. Its application as pedagogical affordance has also been frequently recommended for addressing the need to make stance resources explicit to students in writing instruction (Chang and Schleppegrell, 2011; Crosthwaite and Jiang, 2017; Jou, 2019). To echo this call, a few intervention studies have been conducted to examine the practicality and effects of explicit stance instruction on students’ learning of academic genres. For example, Chang and Schleppegrell (2016) converted the Engagement framework into a concordance tool to scaffold EFL doctoral students’ learning of academic introduction. Results revealed that students gained improvement in stance-taking to accomplish specific rhetorical purposes with more appropriate prosody. Jou (2019) introduced the Engagement metalanguage in second language (L2) writers’ learning of article reviews. Results showed that students exhibited better control over evaluation and expanded their repertoire of stance expressions. Zhang and Zhang (2021b) conducted an Engagement-based writing intervention with Chinese EFL undergraduates and found that students showed improvement in mitigating and integrating source texts by using more varied stance expressions. The positive results in previous studies provide evidence for the effectiveness of an SFL Engagement-assisted writing instruction in improving student writers’ strategic use of stance resources in various academic genres. To date, however, little attention has been granted to the effects of such an instruction on students’ understanding of stance, as well as their more general understanding of academic writing. To test the efficacy of explicit stance instruction, these two aspects should not be neglected as learners’ understandings of stance and writing have affinities with how they convey ideas and integrate sources in writing practice (Zhang and Zhang, 2021a; Huang and Zhang, 2022).

### Perceptions of stance

The essential role of learners’ perceptions in their writing development has been frequently documented in the literature (Mateos et al., 2011; Zhang, 2016; Wette, 2018; Sun et al., 2021; Sun and Zhang, 2022; Teng, 2022). In this study, perceptions of stance refer to students’ attitudes and feelings toward stance expressions. A few interview-based studies on perceptions of stance have been conducted as complementary to linguistic analyses to obtain an in-depth understanding of writers’ stance construction. Studies with experienced writers found that their stance construction in writing were affected by their perceptions of stance which may vary across disciplines (Hyland, 2005) and degrees of professional experience (Yasuda, 2022). Similarly, Morton and Storch (2019) found that when reading PhD multilingual student writing, supervisors’ perceptions of stance were shaped by their disciplines, personal background, and preferences. Zhao and Wu (2022) further found that, in raters’ stance perceptions of EFL essays, students’ perceptions of stance varied and might be affected by raters’ understandings of the effectiveness of certain language features, essay structure, and evaluative criteria.

To date, only a few studies have explored EFL student writers’ perceptions of stance in academic writing. One study conducted by Chang and Tsai (2014) involved interviews with EFL doctoral students from soft sciences and hard sciences. To better communicate with participants, the researchers simplified stance construct into a dichotomised set by referring to the Engagement framework: assertive stance (i.e., *dialogic contraction*) and tentative stance (i.e., *dialogic expansion*). It was found that students mainly discussed stance as a linguistic, rather than a behavioral or cognitive construct. Their understandings were affected by disciplinary conventions and were not compatible with their mature epistemological beliefs. Using a similar method, Chang (2016) further interviewed twelve EFL doctoral students for their perceptions of stance. Results showed that students were generally positive toward assertive claims due to the sense of authoritativeness, and they felt negative toward tentative claims due to their designation of uncertainty and lack of confidence. Zhang and Zhang (2021a) developed and validated a questionnaire based on the Engagement framework examining EFL students’ perceptions of stance. It has been further revealed that students perceived *dialogic contraction* and *dialogic expansion* as independent domains and their perceptions of assertive stance were correlated with the frequencies of certain contractive resources adopted in written texts.

These studies, using either a quantitative or qualitative approach, drew on the Engagement metalanguage to investigate the status quo of students’ perceptions of stance. However, scant attention has been granted to the changes in stance perceptions as a result of instruction. Additionally, a mixed-methods approach is needed in order to have a more robust grasp of students’ understanding.

### Writing beliefs

Writing beliefs are sets of tacit beliefs that a writer holds on what good writing is and what good writers should do (White and Bruning, 2005; Zhao and Zhang, 2022). Building upon Bereiter and Scardamalia’s (1987) models of knowledge-telling and knowledge-transforming, one widely used model distinguishes two types of writing beliefs: *transmissional beliefs* and *transactional beliefs* (White and Bruning, 2005). They are independent of each other and are associated with differences in writing performance (White and Bruning, 2005; Baaijen et al., 2014). Writers holding predominantly transmissional beliefs view writing as a means of transferring information from authoritative sources to the reader and thus tend to reflect limited personal ideas in the text. In contrast, transactional writing beliefs represent the idea that writing is a way to “personally and critically construct the text by actively integrating own thinking into the process” (White and Bruning, 2005, p. 168). Baaijen et al. (2014) further proposed that transmissional beliefs are concerned with the “source of content”; that is, whether the writing should contain authoritative sources or not, while transactional beliefs are about the “process of writing”; that is, whether the writing process involves idea development (p. 82).

The essential role of writing beliefs as a predictor of writing quality has long been acknowledged and supported in various contexts (Mateos and Solé, 2012; Sanders-Reio et al., 2014; Zotzmann and Sheldrake, 2021). Particularly, prior studies have revealed that writing beliefs influence a writer’s affective and cognitive engagement, as well as writer-reader interaction in the writing process (White and Bruning, 2005; Mateos et al., 2011; González-Lamas et al., 2016). For instance, transactional beliefs are found to positively correlate with idea-content development, organization, and voice in writing outcomes (Mateos et al., 2011; Cuevas et al., 2016; Baaijen and Galbraith, 2018), as well as with writers’ audience awareness, self-efficacy, and positive emotions in the writing process (Neely, 2014; Sanders-Reio et al., 2014). In contrast, transmissional beliefs are found to be a negative predictor of writing performance and a writer’s self-efficacy (Sanders-Reio et al., 2014; Zotzmann and Sheldrake, 2021). Some researchers argued that transactional beliefs may be more adaptive in complex writing assignments and that the enjoyment engendered would encourage students to write productively; transmissional beliefs, in contrast, could foster “a mechanical and/or safe, self-protective, and detached approach to writing that entails stringing other writers’ quotes together” (Sanders-Reio et al., 2014, p. 9).

The theoretical assumption and empirical evidence align to support the interaction between a writer’s writing beliefs and stance-taking, that is, how the writer presents ideas, engages readers, and incorporates sources in writing. However, less classroom-based intervention research is conducted, regarding how writing beliefs develop as a result of instruction of stance-taking.

## Methods

Informed by the theoretical framework and prior research, this study was designed in a convergent mixed-methods approach investigating the effects of an SFL Engagement-based instruction on EFL students’ perceptions of stance and writing beliefs in the context of China. Two research questions guided this study.

RQ1: How did the explicit instruction affect students’ perceptions of stance?

RQ2: How did the explicit instruction affect students’ writing beliefs?

### Research context

This study was conducted in a compulsory English academic writing course for Year 3 English major undergradutes during a 4-year program in a northern China university. The objective of this course was to prepare students for their final thesis for the bachelor’s degree by assisting them in acquiring knowledge and writing skills needed for academic argumentation. Two intact classes were involved in this study and were taught by the same instructor who has been delivering the course for more than 3 years.

### Participants

A total of 50 EFL students from the two intact classes participated in this study. The two classes were randomly assigned into the treatment group (*n* = 26) and the comparison group (*n* = 24). The treatment group received an eight-week instruction with the explicit teaching of stance metalanguage based on the SFL Engagement framework, while the comparison group received regular curriculum-based instruction at the same period. All the participants had been studying English for at least 9 years and none of them had learned academic writing before.

Two students from each group were voluntarily invited for a multiple-case study to obtain in-depth information about the effects of writing instruction on students’ perceptions of stance and writing beliefs. They were selected from volunteers using purposive sampling according to two criteria: (1) students with average or good English writing proficiency (2) have a habit of keeping weekly journals or diaries. Table 1 presents the participants’ profiles in the multiple-case study.

**Table 1:** Participants’ profiles in the multiple-case study.

Group	Pseudo-name	Gender	Age	Year of English learning	EAP learning experience
Treatment	Ada	Female	21	9	None
	Danielle	Female	22	9	None
Comparison	Song	Female	21	10	None
	Jing	Female	21	9	None

### Instruments

#### Perceptions of authorial stance questionnaire

This study used Zhang and Zhang’s (2021a) Perceptions of Authorial Stance Questionnaire (PASQ), which was developed and validated in EFL writing contexts in tertiary education that was highly similar to the current research context. The five-point Likert-scale contains 17 items generated based on the SFL Engagement framework, measuring two perceptive intentions toward stance in writing: *preference for dialogic contraction* (9 items) and *preference for dialogic expansion* (8 items). As the participants in this study were advanced undergraduate students studying English as their major, the English PASQ was used. The clarity and readability of the items were first checked by five non-participant students enrolled in the same course. The scale was then piloted with 39 students for reliability using Cronbach’s alpha coefficient. The Cronbach’s alpha values for the two factors were 0.853 and 0.723, indicating satisfactory reliability.

#### Writing beliefs inventory

White and Bruning’s (2005) Writing Beliefs Inventory (WBI) was used to elicit students’ writing beliefs. The 5-point Likert-scale encompasses 19 items and elicits two kinds of writing beliefs: *transmissional beliefs* (6 items) and *transactional beliefs* (13 items). Given that the instrument has been mostly used with first language (L1) student writers of English in previous studies (e.g., Mateos et al., 2011; Baaijen et al., 2014; Neely, 2014; Cuevas et al., 2016), the reliability of the English questionnaire was examined with EFL students using the Cronbach’s alpha coefficient. The validation process involving 191 EFL students resulted in the removal of two items and the revised questionnaire showed acceptable reliability (Cronbach’s α for the whole scale was 0.722 and for the two factors was 0.619 and 0.715, respectively).

#### Semi-structured interviews with text judgment

Semi-structured interviews were used in the multiple-case study to elicit in-depth information about participants’ perceptions of stance and writing beliefs. An interview is a qualitative inquiry method that involves a one-to-one conversation to obtain participants’ interpretations of the target phenomena from their perspective (Dörnyei, 2007). A semi-structured interview is composed of a set of pre-prepared open-ended questions providing guidance and direction for the participant (Creswell and Creswell, 2018).

In this study, each interview included three parts (see Appendix A for the interview protocol). The first part inquired about participants’ background information and previous learning experience. The second part elicited participants’ views on academic writing. Informed by previous studies (e.g., Chang and Tsai, 2014; Chang, 2016), the third part involved text judgment to yield participants’ perceptions of stance. Two versions of the same text were provided that are different in the use of stance resources. Participants were asked to articulate their perception of each text and personal preference. The two texts for interviews prior to and after the instruction were selected from the introduction sections of two published articles^1^ in the field of social sciences. Each text was rendered into two extreme versions with regard to stance (an assertive version and a tentative version), based on the dichotomy of *dialogic contraction* and *dialogic expansion* in the Engagement framework. Table 2 presents examples of the two versions of a text, with key stance markers italicized.

**Table 2:** Examples of two versions of text in interviews.

Assertive version	Tentative version
Academic achievement reflects the capacity to attain learning goals included in the school curricula and is *clearly* related to important outcomes. There is increasing evidence that the capacity to solve typical academic problems (e.g., in mathematics and reading) *can* predict future educational or academic outcomes. The fact that achievement is *strongly* related to life outcomes is not surprising. In fact, academic achievement tests involve intelligence, extensive reasoning, and problem-solving capacity. It *must* be noted that some cognitive factors are related to academic achievement.	Academic achievement reflects the capacity to attain learning goals included in the school curricula and is related to important outcomes. There is *some* evidence that the capacity to solve typical academic problems (e.g., in mathematics and reading) *tends to* predict future educational or academic outcomes. The fact that achievement is *somehow* related to life outcomes is not surprising. In fact, academic achievement tests involve intelligence, extensive reasoning, and problem-solving capacity. It *should* be noted that some cognitive factors are related to academic achievement.

#### Reflective journals

Participants of the multiple-case study were also asked to keep weekly reflective journals during the period of writing instruction. A reflective journal is a valid way of eliciting a first-person account of a language learning experience; it provides students with the opportunity to reflect on their beliefs in a natural way, from which researchers can access time-related development or fluctuation within participants, as well as their responses to certain stimuli (Dörnyei, 2007). In this study, participants were prompted to reflect on their experiences concerning academic writing, stance-taking, and writing instruction received. See Appendix B for the reflective journal prompt.

### Writing instruction

The writing intervention was implemented within a required course on academic writing for Year 3 English-major undergraduates. The course lasted for 16 weeks in total and 8 weeks (45 min per week) were allotted to learning academic argumentation. During this 8-week, the treatment group received the intervention with stance expressions explicitly taught and valued based on the SFL Engagement framework. The instructor illustrated each stance type and its dialogical functions and discursive effects, with the help of which the valued qualities of academic writing were made transparent to students. The technical language in the Engagement framework was substituted by a series of graduated terms to better facilitate students’ noticing and acquisition (see also Chang and Schleppegrell, 2016; Zhang and Zhang, 2021b). For instance, monoglosses were introduced as *“non-argumentative*” stance. Resources for dialogic contraction and expansion were introduced as “*high-argumentative*” and “*low-argumentative*,” respectively. During these sessions, reading tasks were provided using sample introductions of published articles, in which students were guided to analyze stance deployment and then evaluate their stance-taking in writing. The academic introduction was selected because it is distinctively featured with the author’s use of stance resources as they present opinions and construe argumentation (Sawaki, 2014). At the same period, the comparison group received curriculum-based instruction on academic argumentation without explicit provision of stance metalanguage.

### Data collection procedures

Participants in both groups were asked to complete two questionnaires (PASQ and WBI) prior to and immediately after the writing intervention. The questionnaires took each participant approximately 20–30 min to complete.

Four participants were voluntarily recruited to participate in the multiple-case study in which semi-structured interviews were conducted prior to and immediately after the writing intervention. Each interview was conducted in an empty classroom with each participant individually and lasted approximately for 30 min. In the interview, the research purpose was explained and the participants were assured that they were entitled to refuse to answer any questions. Questions in the interview protocol were asked in L1 Chinese as preferred by the participants, and they were entitled to respond in either L1 or L2 English. Participants were also informed that the interviews were audio-recorded. The questionnaires that were obtained from the four participants were not included in the study of group performance to prevent data contamination, as taking part in interviews might influence how these students respond to questionnaires.

In the multiple-case study, the four participants were also asked to keep weekly reflective journals in either L1 or L2. Their journal writing was prompt-driven with each journal entry requiring approximately 20 min to complete. Participants were required to submit the journal electronically within 2 days after the writing course every week, except for Weeks 1 and 8 to avoid closing in time for interviews; 24 journals were thus collected in total. All the participants were provided with a pseudonym as part of the research project to protect their identity and guarantee confidentiality.

### Data analysis

#### Questionnaires

Quantitative data collected from the two questionnaires were first screened and cleaned. After the examination of normality, independent-samples *t*-tests were used to investigate whether there are differences in students’ perceptions of stance and writing beliefs between the two groups prior to and after the writing intervention. Paired-samples *t*-tests were applied to explore the within-group differences after the writing instruction. Cohen’s *d* was used to measure the effect size of the significant difference (small = 0.2; medium = 0.5; large = 0.8) (Cohen, 1992).

#### Interviews and reflective journals

All the audio-recordings of interviews were transcribed and translated into English. We analyzed the interview transcripts and reflective journals through a thematic approach combining inductive and deductive analysis (Braun and Clarke, 2006). Firstly, we iteratively read the interview transcripts, particularly the second part, with continuous reference to the previous literature and the research questions to identify themes in students’ reported writing beliefs. For instance, under the theme of *transmissional beliefs*, we further identified two subthemes: *information transmission* and *authoritative evidence*; under the theme of transactional beliefs, two subthemes were identified: *active engagement* and *critical evaluation of others’ views*. We then deductively analyzed the transcripts of the third part of the interview to identify participants’ perceptions of stance. We focused on perceptions of assertive stance (*dialogic contraction*), perceptions of tentative stance (*dialogic expansion*), and participants’ preferences when comparing the two stance categories. A deductive analysis of reflective journals was then conducted based on the themes and subthemes identified in the interview transcripts. To ensure the reliability of the data analysis, we analyzed the data independently and then collaboratively. Disagreement was resolved through discussion. Within-group and between-group comparisons were then conducted to synthesize the findings to obtain a comprehensive description of the impacts of writing instruction on participants’ reported writing beliefs and perceptions of stance.

## Results

### Findings from questionnaires

This section reports quantitative findings from questionnaires. Shapiro–Wilk tests and histograms showed that the variables of perceptions of stance and writing beliefs were in normal distribution.

#### Within each group

A series of paired-samples *t*-tests were applied to compare the writing beliefs and beliefs about authorial stance in the pre-test and post-test within each group. Table 3 demonstrates the descriptive statistics and results of paired-samples *t*-tests. In terms of writing beliefs, the mean score of transactional beliefs of the treatment group marginally increased approaching the level of significance with a small effect size (*p* = 0.075; Cohen’s *d* = 0.381), while the mean scores of transmissional beliefs and scores of the comparison group remained statistically unchanged. This implies that the 8-week explicit instruction only had a marginal effect, if any, on students’ transactional writing beliefs. In terms of perceptions of stance, both the mean scores of dialogic contraction and dialogic expansion in the two groups did not show significant changes, indicating that the change in students’ perceptions of stance was not visible after 8 weeks of instruction in both groups.

**Table 3:** Descriptive statistics and paired samples *t*-tests of writing beliefs and perceptions of stance within each group.

Group	Variables		Pre-test		Post-test		*t*	*p*	Cohen’s *d*
			M	*SD*	M	*SD*			
Treatment group (*n* = 24)	Writing beliefs	TM	2.86	0.68	3.02	0.57	1.163	0.257	0.237
		TA	3.62	0.42	3.80	0.33	1.868	0.075	0.381
	Perceptions of stance	DC	3.51	0.36	3.53	0.40	0.131	0.897	0.027
		DE	3.23	0.47	3.43	0.42	1.435	0.165	0.293
Comparison group (*n* = 22)	Writing beliefs	TM	2.88	0.63	2.85	0.61	−0.182	0.857	0.039
		TA	3.68	0.35	3.63	0.27	−0.594	0.559	0.127
	Perceptions of stance	DC	3.47	0.60	3.48	0.38	0.121	0.905	0.026
		DE	3.17	0.29	3.27	0.30	1.752	0.202	0.281

TM, Transmissional beliefs; TA, Transactional beliefs; DC, Dialogic contraction; DE, Dialogic expansion.

#### Between-groups comparisons

A series of independent-samples *t*-tests were conducted to investigate whether there were statistical differences between the two groups in the post-test. Results revealed no significant difference between the two groups with regard to writing beliefs and perceptions of stance: transmissional beliefs, *t*(44) = 0.937, *p* = 0.354, Cohen’s *d* = 0.268; transactional beliefs, *t*(44) = 1.973, *p* = 0.055, Cohen’s *d* = 0.649; dialogic contraction, *t*(44) = 0.410, *p* = 0.684, Cohen’s *d* = 0.124; and dialogic expansion, *t* (44) = 1.449, *p* = 0.154, Cohen’s *d* = 0.524. Although not statistically significant, students from the treatment group reported a marginally higher level of transactional beliefs (*M*. = 3.80, *SD* = 0.33) than the comparison group (*M* = 3.63, *SD* = 0.27) and dialogic expansion (*M* for the treatment group = 3.43, *SD* = 0.42; *M* for the treatment group = 3.27, *SD* = 0.30) in the post-test with a medium effect size. This implies that compared with the curriculum-based instruction, explicit stance instruction had a marginal effect on students’ transactional writing beliefs and perceptions of dialogic expansion, though the effects were not statistically significant.

### Findings from the multiple-case study

This section reports findings from the multiple-case study drawing on qualitative data from semi-structured interviews and reflective journals. Following the proposed order of questions in interviews, we first report results on students’ writing beliefs and then perceptions of stance. Each theme and subtheme are illustrated, respectively, in the following sections with excerpts of students’ responses in interviews. Extracts from reflective journals are used to complement the findings of interviews to achieve a better understanding of students’ reported writing beliefs and perceptions of stance.

#### Students’ reported writing beliefs

Students’ reported writing beliefs revealed two major themes: *transmissional writing beliefs* and *transactional writing beliefs*. Two subthemes were identified in transmissional beliefs: *information transmission* and *authoritative evidence*; two subthemes in transactional beliefs: *active engagement* and *critical evaluation of others’ views*. Table 4 provides an overview of identified themes in each participant’s reported writing beliefs in the interviews. Results showed that participants from the two groups similarly articulated transmissional writing beliefs prior to and after the writing instruction, while changes can be identified in transactional beliefs, particularly for participants in the treatment group.

**Table 4:** Themes in students’ self-reported writing beliefs in interviews.

Themes		Treatment group				Comparison group			
		Ada		Danielle		Song		Jing	
		Pre	Post	Pre	Post	Pre	Post	Pre	Post
Transmissional writing beliefs	Information transmission	√	√	√	√	√	√	√	√
	Authoritative evidence	√	√	√	×	√	√	√	√
Transactional writing beliefs	Active engagement	×	√	√	√	√	√	×	×
	Critical evaluation of others’ views	×	×	×	√	×	×	×	√

“Pre” means prior to writing instruction. “Post” means after writing instruction. “√” indicates that students expressed views concerning the subtheme. “×” means that students did not express views on the subtheme.

##### Transmissional writing beliefs

###### Prior to writing instruction

The theme of transmissional writing belief includes two subthemes: *information transmission* and *authoritative evidence*. The first subtheme, *information transmission*, refers to the belief that academic writing is a knowledge-telling process and the purpose of writing is to provide information to readers. The second subtheme, *authoritative evidence*, assumes that academic writing should involve authoritative evidence or sources. All the participants similarly articulated the two subthemes prior to the writing intervention. For instance, Excerpt 1 showed Ada’s perspective on academic writing as an information-transmitting process and academic texts as a repository of knowledge.

Excerpt 1 “Firstly, academic writing is to illustrate one thing, provide other people some information, informational knowledge or methodological knowledge.” (Ada, TPr^2^)

Excerpts 2–4 demonstrate that all the participants similarly emphasized the importance of including authoritative evidence as support for argumentation. In their opinion, convincing arguments should involve authoritative sources, such as data, citations, resources in professional journals, and facts. In Excerpt 3, Danielle further expressed that substantial data and citations in writing could contribute to the persuasiveness of argumentation.

Excerpt 2 “The opinion should be based on firm evidence, either data or citations. It should be illustrated logically.” (Ada, TPr)

Excerpt 3 “(For good academic writing) we should look at its wording, arguments and format. And whether the data cited are authoritative or not. … Try to use more citations or data. … I think I should provide more facts so that my writing can be more convincing.” (Danielle, TPr)

Excerpt 4 “You can write whatever you want for informal writing. But for thesis, you need to refer to a lot of materials, such as those in influential journals.” (Jing, CPr)

###### After writing instruction

After the writing instruction, all four participants continued to articulate their belief in writing as a means of information transmission, as shown in Excerpts 5–7.

Excerpt 5 “Academic writing is to introduce information or deliver knowledge. This is the purpose.” (Ada, TPo)

Excerpt 6 “You did some research and then you want to communicate to others, you have to convey a certain information.” (Danielle, TPo)

Excerpt 7 “I think academic writing should be preciseness in both structure and contents because it aims to convey accurate information.” (Danielle, 6^th^ Journal)

Additionally, all the participants, except Danielle, expressed similar emphasis on authoritative evidence in argument development, to prevent potential challenge or conflict from readers, as shown in Excerpt 8.

Excerpt 8 “It should have a viewpoint, and most importantly have examples of facts. That is, it should have examples to prove its view.” (Ada, TPo)

In summary, participants from both groups articulated similar transmissional beliefs that academic writing is an information-transmission process that relies, predominantly, on authoritative evidence. This view remained largely unchanged after the writing intervention.

##### Transactional writing beliefs

###### Prior to writing instruction

The theme of transactional writing belief includes two subthemes: *active engagement* and *critical evaluation of others’ views*. The first subtheme, *active engagement*, assumes that a writer should actively incorporate his or her thinking into writing or actively engage with putative readers. The second subtheme, *critical evaluation of others’ views*, refers to the belief that a writer should critically evaluate and incorporate others’ views in the writing process. Prior to the writing intervention, one participant from each group, Danielle and Song, similarly exhibited the first subtheme, while none of the four participants articulated the second subtheme.

As shown in Excerpts 9 and 10, Danielle and Song similarly mentioned that writers should actively integrate their thinking when constructing arguments. They also claimed that active engagement, or expression of views, should include facts or examples as supporting evidence, which reflects transmissional beliefs. It can be noted that although they expressed a transactional understanding of the writing process, they tended to incorporate personal views and actively engage with readers in a transmissional way. This reflects Baaijen et al.’s (2014) dual-process hypothesis that transmissional beliefs are concerned with the “source of content,” while transactional beliefs are about the “process of writing” (p. 82).

Excerpt 9 “Firstly, it (academic writing) should have a research direction and then the writer should discuss the direction with his or her own viewpoints. … The purpose of arguments is to convince others and support your own ideas as well. … Good arguments should be consistent with your own ideas. And you should find some corresponding reasons, such as facts or examples, or data, to support your ideas.” (Danielle, TPr)

Excerpt 10 “The purpose of argumentation is to arouse readers’ interest. They should know what your paper is about. …Firstly, you should present facts to let other people know the issue. And then you can express your viewpoint about this.” (Song, CPr)

###### After writing instruction

After the writing intervention, the two groups reported differently on both subthemes of transactional beliefs. Regarding the subtheme of *active engagement*, both of the participants from the treatment group reported the need for a writer’s active involvement in argument writing; they further exhibited a clear awareness of stance that was qualitatively distinct from their previous views. For instance, in Excerpt 11, Ada clearly articulated that a writer should actively integrate his or her own views to convince putative readers. An overt recognition can be noticed of the usefulness of applying various types of stances for specific purposes in argument construction.

Excerpt 11 “The purpose of an argument is to convince readers. … In the process of writing, an objective voice should be used to state facts and a strong voice should be used to express the writer’s opinion to convince readers. … Good argument is to use low-argumentative when talking about others’ views. Then readers will feel, er, something is not right. Then when they read the writer’s view, they will feel it quite strong and certain.” (Ada, TPo)

In Excerpt 12, Danielle further showed self-awareness of the changes in her understanding. Assertive stance was introduced as “high-argumentative” in the writing intervention. Danielle had noticed her previous transmissional tendency to include facts for argument construction, and she then expressed her recognition of the importance of the use of stance to achieve the goal. A transition in writing beliefs and enhanced stance awareness can be identified.

Excerpt 12 “When we wrote argumentations before, we were used to stating a lot of facts to prove that our point of view is convincing. But academic writing is different. … And high-argumentative sentence must be used to make argumentation persuasive.” (Danielle, 6th Journal)

One participant from the comparison group, Song, also articulated her views on active engagement after the writing instruction. But as shown in Excerpt 13, her perspective was essentially unchanged when compared to her views prior to the instruction (Excerpt 10). While she mentioned a writer’s active role in integrating personal opinion in arguments, her focus remained on the reader’s understanding. In her view, the purpose of argumentation should be on the successful transmission of content issues to readers, such as “purpose,” and “positive and negative sides of the issue,” and that the way to achieve effective arguments was an either direct or indirect presentation of the viewpoints. Thus, although she expressed her awareness of active engagement, her way of achieving the goal of convincing arguments remained transmissional, as she responded before the writing instruction.

Excerpt 13 “The purpose of argumentation is to let readers understand your writing, what you are doing, your purpose. … Effective arguments can be achieved in two ways. First, you can directly say your opinion about the issue. Second, you can be indirect and discuss positive and negative sides of the issue.” (Song, CPo)

In terms of the second subtheme, *critical evaluation of others’ views,* one participant from each group, Danielle and Jing, reported relevant views but with varying degrees of stance awareness. In Excerpt 14, Danielle pointed out that writers need to critically evaluate previous studies to build a solid foundation for their own opinions in academic writing. She further exhibited stance awareness by articulating that tentative stances should be adopted when referring to others’ views.

Excerpt 14 “For the same topic, other people may have already done some research. So in such circumstances, if you want to attract readers, you must summarize previous studies and point out their shortcomings. And then you can express your viewpoint. … Then use tentative stances to cite others’ views.” (Danielle, TPo)

Jing from the comparison group similarly expressed the necessity of establishing arguments based on critical evaluation of previous views, as shown in Excerpt 15. The difference between her view and Danielle’s lies in that she did not overtly express an awareness of stance.

Excerpt 15 “If I want to express a view, I can firstly use others’ views that are different from my own, and then go to my own opinions. Or I can use similar examples from others, such as similar research, and then I will say what I have done further. … I can directly express my view, and then to say the differences of my study and other studies.” (Jing, CPo)

In summary, prior to the writing instruction, participants in both groups similarly acknowledged the writer’s active engagement to express personal views in writing but with a transmissional tendency. They also appeared to be unaware of the need to critically evaluate others’ views. After the writing intervention, qualitative differences can be noticed between the two groups. The treatment group participants expressed the need for active engagement and critical evaluation of others’ views in academic writing and explicit awareness of stance-taking for establishing arguments. Although the comparison group participants expressed an increased awareness of critical evaluation of previous views, their understanding mainly focused on writing content without exhibition of stance awareness.

#### Students’ reported perceptions of stance

The analysis of participants’ perceptions of stance focused on three themes: *perceptions of assertive stance*, *perceptions of tentative stance*, and *stance preference*. Results showed that at different times, participants from both groups used similar linguistic expressions to describe how they perceive assertive and tentative stances, respectively. With regard to stance preference, participants from both groups exhibited a similar preference for tentative stances for their own writing experience. However, after the writing intervention, the treatment group participants showed a preference for assertive stances, while the comparison group stayed with a tentative preference.

##### Perceptions of assertive stance

Results showed that the participants from both groups deployed similar words to depict assertive stance, as shown in Table 5. At different times, participants most frequently described assertive stances from an epistemic domain by using the words like “*strong*,” “*certain*” or “*sure*.” They paid attention to the strengths of claims and levels of certainty when they encountered assertive stances. The depiction of assertive stance as “*absolute*,” rather than revealing a dialogic understanding, reflects participants’ reluctance to be extreme and that they felt safer being neutral to avoid contradiction.

**Table 5:** Participants’ perceptions of assertive stance.

Groups	Participants	Pre-interview	Post-interview
Treatment group	Ada	Because in each sentence, there are some words like “strongly”, and “clearly.” They make me feel that the writer is very *certain* about the issue. As a reader, I am more likely to *trust* assertive text with a *strong* voice.	In the assertive text, … the voice is very *certain* and *strong*. It makes the reader feel like, “Yes, it is right.”
	Danielle	I feel that it is too *absolute*. There is nothing absolutely right or wrong.	In the assertive text … these words show that the writer is very *sure* about the research and his/her own opinion, and convey a *certain* view to readers.
Comparison group	Song	The assertive text is surely more convincing. Because the words in it, no…it cannot be called convincing, the words in it are quite *absolute*. Because the text seems to remain the writer’s… uh… assure that this is the writer’s personal opinion.	Because there is nothing to be definitely correct. … The assertive text is very *absolute*, like “undoubtedly.”
	Jing	The writer of the assertive text seems quite *sure* about what he says. I feel the writer is quite *sure* and knows the research field quite well.	I think, usually when a writer uses the words like “must”, there are always other things that are contradictory. People can always find something to argue with him.

However, a perceived change could be noted in Danielle’s journal toward assertive stance in academic writing amid the writing intervention, as shown in Excerpt 16. her attitude toward assertive stance had slightly changed from a negative perception of assertive stance as being absolute, to considering its usefulness in academic writing.

Excerpt 16 “In the past, I thought that it is unnecessary for academic writing to write in a high-argumentative tone because it is too exaggerated. However, after the last class, I thought high-argumentative stance is also really useful in academic writing.” (Danielle, 3rd Journal)

##### Perceptions of tentative stance

Table 6 lists examples of participants’ perceptions of tentative stance. Results showed that participants in both groups did not articulate differently when describing tentative stances. The expressions that were used frequently were “*not strong*,” “*uncertain*,” and “*not absolute*.” It can thus be noted that participants perceived tentative stances as the opposite side of assertive stances. They similarly focused on the strengths of claims and levels of certainty that the stance features could bring to the text. It could also be noticed that, at different times, participants mentioned that a tentative stance could “*leave some space*” for others and avoid being absolute. This thus appears to be a passive decision for participants to avoid challenges or handle unfamiliar content issues, instead of an active strategy to establish a dialogic space with readers.

**Table 6:** Participants’ perceptions of tentative stance.

Groups	Participants	Pre-interview	Post-interview
Treatment group	Ada	When I read the tentative text, the feeling is just so-so, *not* as *strong* as the assertive text. The tentative text makes the reader think. The words in it, such as “somehow related to life outcomes,” make me consider the possibility in my life.	In the tentative text, the same sentence is written as “are probably part of.” So the reader also feels like, “OK, maybe like this.” Then, when the writer refers to others’ pinions, the reader further feels *uncertain*…
	Danielle	The tentative text is *neutral*. It has its view and also provides readers with some space to express their own opinions. I do not think the tentative text is weak. I think its voice is quite suitable for expressing opinions. Generally, the formal text is like this, not to show the writer a lot, *not* to use *too strong* voices.	The tentative text is *uncertain*. The words, like “according to somebody” or “suggests,” expressed opinions that are not so certain. So for the field that you are not so familiar with, you can *leave some space* for others to discuss.
Comparison group	Song	The words in the tentative text make me feel that the writer is *not so sure* about his or her own views. … It makes me feel that other people can push your ideas over with other evidence.	Because there is nothing to be definitely correct. The tentative text is better because the writer expresses his/her own views *without being absolute*.
	Jing	The writer in the tentative text is *not so certain*, such as he uses “it could be suggested”, and “seem to.” I feel his view is not so sure. It *leaves some space* for readers and does *not* say things in an *absolute* way.	I think the tentative text is better. When I wrote previously, for example, I used “will” in my writing. Then the teacher would change it with another word that is *more mild* and euphemistic.

##### Stance preference

###### Prior to writing instruction

Prior to the writing intervention, all the participants from both groups, except Jing from the comparison group, explicitly expressed a preference for tentative stances in their writing. For instance, in Excerpt 17, Danielle explained that her preference for tentative stance was relevant to her negative feeling toward assertive stance. As reported earlier, participants thought that assertive stance can strengthen the claims (e.g., using expressions “*certain*” or “*sure*”) but also raise potential challenges (e.g., “*absolute*”); the latter effect apparently outweighed the former when Danielle considered deploying stance in her own writing. Therefore, she considered assertive stance as being over extreme and regarded tentative stance as a better, or safer choice to deploy in writing.

Excerpt 17 “When I’m writing, I tend to write like the tentative text. That is, not to be too absolute. The assertive text looks too absolute. It makes me feel like that there is only one way to go, only this direction, no other ways.” (Danielle, TPr)

Different from the other three participants, Jing expressed a preference for assertive stance in her own writing. However, as shown in Excerpt 18, she explained that it was exam-oriented and otherwise her personal preference was to write tentatively. Similar to other participants, Jing’s personal preference for the tentative stance was rooted in its less-absolute nature. She further mentioned that she was taught to use assertive stances in English writing, the reason for which she did not fully understand. With the teacher’s constant emphasis, she had to apply more assertive stances for test preparation. Jing appeared not to consider these linguistic expressions as stance markers and only applied them as taught without understanding why they might contribute to convincing arguments.

Excerpt 18 “But sometimes I also feel that the tentative text is more objective. It leaves some space for readers and does not say things in an absolute way. But I was taught in class that I should state things like the assertive text. … So sometimes I feel confused. … Now, I may write like the assertive text. But previously I would write like the tentative text. Because of the teacher’s emphasis in class, … I will use words like ‘strongly’, and ‘significantly’. … This is all for the preparation for the TEM4 test.” (Jing, CPr)

Participants’ responses showed that when considering stance preference for their own writing, they weighed the potential challenges more than the certainty that assertive utterances might bring so they resorted to tentative utterances for a more neutral or safer choice that could reduce such risks. It thus can be manifested that their stance preference was a passive or conservative choice.

###### After writing instruction

After the writing instruction, participants from the two groups reported different stance preferences. Both participants from the treatment group exhibited a changed preference for assertive stance. In Excerpts 19–21, both Ada and Danielle clearly stated their preference for assertive stance in their writing. They valued the epistemic certainty brought by assertive stance over the worry of extreme or absoluteness, which was saliently different from their previous preference. Ada’s words further showed her increased self-confidence when confronting potential challenges from readers. In addition, they considered deploying a mixture of assertive and tentative stances to fulfil various rhetorical purposes for constructing convincing arguments. Both of them articulated the tendency to use assertive stances for expressing their own views and to use tentative stances for introducing views from sources. It can be noted that they exhibited a more active decision of stance use with the critical judgment of the functions and usefulness of different stance types, which was markedly different from their previous understanding.

Excerpt 19 “I think I will use assertive stance to express opinions in my writing. … and use low-argumentative voice when referring to others’ views. Because I think as a writer, you should make readers feel certain when expressing your own opinions. If you are not certain, other people will not believe what you write. If you think that other people may challenge your ideas, you use low-argumentative voices to provide some space for discussion. But I think, you can be certain and other people can also argue with you.” (Ada, TPo)

Excerpt 20“After the writing course, I think I will write more like the assertive text. I will be certain when expressing my own opinions. And I may also add some uncertain views when reviewing previous studies.” (Danielle, Tpo)

Excerpt 21 “And the mixture of two stances is also the way to make our academic writing convincing. Because the reader can be convinced by high-argumentative statement and also can know the author’s preciseness.” (Danielle, 3rd Journal)

After the writing instruction, Song and Jing from the comparison group expressed an unchanged preference for tentative stances in their own writing, with similar reasons as previously. As evidenced in Song’s words in Excerpt 22, they held a negative attitude toward assertive stance due to its being over absolute or challenge-raising.

Excerpt 22 “Because there is nothing to be definitely correct. The tentative text is better because the writer expresses own views without being absolute. … I tend to be like the tentative text when I’m writing.” (Song, CPo)

To summarize, participants from both groups reported a preference for using tentative stances in their own writing prior to the writing intervention; their preference was largely a passive response caused by a negative attitude toward assertive stance. After the writing instruction, stance preference of the comparison group participants was unchanged, as were their underlying reasons; whereas the treatment group participants changed to show a preference for assertive stance and were inclined to use both types of stance for various rhetorical purposes in writing. A more active choice with critical judgment in stance use was observed.

## Discussion

### Effects of intervention on EFL students’ perceptions of stance

Results from questionnaires indicate no statistically significant changes in perceptions of stance after the period of writing intervention for either group. This was triangulated by the students’ descriptive reports in the multiple-case study which suggested that their perceptions of the dichotomised stance types were largely unchanged. These results may be due also to insufficient time for any indication of significant changes in students’ perceptions anchored in L1 culture, prior learning history and experiences, genre, or context, to emerge (Silva and Nicholls, 1993; Hyland and Milton, 1997; Sanders-Reio et al., 2014). Additionally, after the writing instruction, there were no salient changes in students’ tendency to describe stance in the epistemic domain, especially using polarized descriptors (e.g., *strong* vs. *not strong*, *certain* vs. *uncertain*) that are concerned with the strengths of claims. This corroborates Chang’s (2016) finding that EFL doctoral students most frequently conceptualized stance as a linguistic construct referring to the strengths of claims, the extent of precision, or promoting research. Chang (2016) argued that students lacked a robust understanding of stance and their beliefs were narrow in scope. Similar to the multiple-case study, students retained the understanding of stance as primarily a linguistic construct and rarely discussed it from other perspectives, such as a dialogic perspective (Chang and Tsai, 2014; Yasuda, 2022). This finding suggests that students still possessed limited language to explain assertive and tentative stance options though they were explicitly provided with stance metalanguage in instruction. It could be attributed to the insufficient time of instruction for significant changes in students’ expanded metalanguage to become apparent, which may be shaped by their language background and personal histories (Sanders-Reio et al., 2014; Morton and Storch, 2019; Teng, 2022).

Probably the starkest difference revealed by the multiple-case data was in students’ stance preference and underlying motivation. The treatment group participants reported a shift of preference for assertive stance over tentative stance if they wrote after the writing intervention, which is consistent with Chang’s (2016) finding that EFL doctoral students exhibited more positive feelings toward assertive stance compared with tentative stance. In contrast, the comparison group students retained their preference for tentative stance at different times. The preference shift of the treatment group students can be explained by their reported motivation, which changed from avoiding potential challenges from readership to actively expressing authorial opinions. This resonates with Zhang and Zhang’s (2021a) finding that EFL students perceived assertive and tentative stances from different perspectives (e.g., writer perspective vs. reader perspective), rather than with varying degrees of dialogic interaction. It is probable that the writing intervention provided students with opportunities to actively monitor and evaluate stance-taking in their own writing, and thus increased their author-oriented motivation and uptake of self-confidence as an author.

In addition, there is an intention articulated by the treatment group participants of using a mixture of assertive and tentative stances to construct convincing arguments for specific rhetorical purposes. This suggests that students tended to deploy different stance types more purposefully, indicating that they were becoming more confident in why and how to use stance resources. The finding, in line with previous contentions (e.g., Miller et al., 2014; Ryshina-Pankova, 2014; Chang and Schleppegrell, 2016; Jou, 2019), provides further evidence for the potential of an explicit approach of stance teaching in encouraging students to use a wider range of stance options. The findings suggest that students’ understanding, especially of the functions and effects of various stance types, improved, which may also have increased their self-confidence in task performance.

## Conclusion

This study examined the effects of explicit stance instruction based on the SFL Engagement framework on EFL students’ perceptions of stance and writing beliefs. It is evident in the findings, particularly qualitative findings, that the instruction was effective in enhancing students’ transactional understandings of writing as dialogic communication and intention of active engagement in the writing process. Empowered to recognize the usefulness of stance markers, students were inclined to adopt a wider range of stance options for various rhetorical purposes in writing practice. This study makes a contribution by applying the SFL Engagement framework to EFL undergraduates’ learning of academic writing and lends support to the previous supposition that the framework can serve as a pedagogical affordance for enhancing novice writers’ stance awareness (Chang and Schleppegrell, 2016; Jou, 2019; Zhang and Zhang, 2021a). Additionally, we would further posit based on our findings that more than awareness-raising, such an instructional affordance can also promote students’ uptake of authorial responsibility and self-confidence in view-expressing in academic writing.

Several pedagogical implications can be drawn from the current findings. Firstly, it can be noted that students were not sufficiently aware of the audience and stance-taking prior to the writing instruction. There is thus a necessity to highlight in instruction that a writer’s meaning-making choices are affected by both the writer’s intention and readers’ expectations. And stance expressions are a viable tool to address the complex relations involved in writer-reader interaction. For instance, when analyzing sample texts, students should be explicitly guided and trained to identify valued stance-taking features and reflect on their rhetorical functions. In addition, given that students possessed limited repertoire when describing assertive and tentative stances both before and after the instruction, it is suggested that a longer period of stance learning should be granted to consolidate students’ awareness and use of stance metalanguage. Thirdly, instructors are suggested to include stance-taking in the assessment criteria for students’ writing performance. This may help students visibly notice the appropriateness of their stance endeavors and learn about stance use more mindfully.

This study possesses a few limitations. Firstly, given that only qualitative changes drawn from four participants were observed, caution should be taken in generalizing the findings and effects of the Engagement-based instruction. Large-scale studies are thus recommended to measure the possible changes in students’ understanding of stance. Additionally, the multiple-case study elicited students’ understanding from a reader’s perspective through text judgment in the interviews. It is thus unclear whether students understand stance-taking differently from the perspective of an author. Further studies are recommended to comply with reflective interviews with students’ texts (e.g., discourse-based interviews) to explore their understandings from a writer’s perspective. Thirdly, this study examined only the short-term effects of explicit stance instruction. As a long period may be needed for psychological factors to evolve (Dörnyei and Ushioda, 2013), future intervention studies could be conducted to examine the long-term effects of instruction to observe the development and maintenance of students’ understanding of stance and writing, which can provide richer data on, and further insight into, the effectiveness of explicit stance instruction based on the SFL Engagement framework.

## Data availability statement

The original contributions presented in the study are included in the article/supplementary material, further inquiries can be directed to the corresponding author.

## Ethics statement

The studies involving human participants were reviewed and approved by the University of Auckland human ethics committee. The patients/participants provided their written informed consent to participate in this study.

## Author contributions

LZ and LJZ conceived and designed the study. LZ collected and analyzed the data and drafted the manuscript. LJZ finalized it for submissions as the corresponding author. All the authors revised and approved the manuscript.

## Funding

This work is funded by an Ocean University of China Start-up Research Grant (No. 862101013219) awarded to LZ.

## Conflict of interest

The authors declare that the research was conducted in the absence of any commercial or financial relationships that could be construed as a potential conflict of interest.

## Publisher’s note

All claims expressed in this article are solely those of the authors and do not necessarily represent those of their affiliated organizations, or those of the publisher, the editors and the reviewers. Any product that may be evaluated in this article, or claim that may be made by its manufacturer, is not guaranteed or endorsed by the publisher.

## Footnotes

^1^The two published articles used in interviews: Prior to the instruction: Giofrè et al. (2017). After the instruction: Nordmark (2017).

^2^TPr = Treatment group, Pre-interview. In the following extracts, TPo = Treatment group, Post-interview; CPr = Comparison group, Pre-interview; CPo = Comparison group, Post-interview.

## Appendix A Interview protocol

**Part I: Background Information 背景信息**

1. Have you taken any English academic writing classes before?
  你是否上过关于英语学术写作的课程?
2. Have you ever written a piece of English academic writing?
  你是否有英语学术写作的经历?
3. What’s your2 academic interest?
  你感兴趣的研究方向是什么?

**Part II: Understanding of Academic Writing 关于学术写作的理解**

1. What is a piece of academic writing?
  你认为什么是学术写作?
2. What do you think successful academic writing should look like?
  你认为成功的学术写作是什么样子的?
3. What is the purpose of arguments in academic writing?
  学术写作中论述的目的是什么?
4. What is an effective argument in academic writing?
  在学术写作中，怎样的论述是有效的?
5. How should the author express his or her own opinion in the academic writing?
  在学术写作中，作者应该如何发表自己的看法?

**Part III: Text Reading and Response 文本阅读和反馈**

(Students read two versions of an academic introduction 阅读两个版本的学术论文引言)

1. Which one do you think is more convincing?
  你认为哪个更有说服力?
2. What is your opinion about the differences of the two texts?
  你认为两篇引言有什么不同?
3. How would you express your view in your own academic writing?
  你在学术写作中会如何表达自己的观点?

## Appendix B Reflective journal prompts

Instruction: In the journal, you can write anything related to the following aspects. There is no word limit. You can write either in English or in Chinese.

1. What did you learn in the English writing class this week? What is useful for your English writing?
2. Your opinions, attitudes or feelings about English writing or academic writing;
3. Problems you encounter in your English writing;
4. How to make your arguments sound convincing;
5. How to express your own opinions in English writing;
6. What do you want to learn more about English academic writing?
7. What suggestions you get from teachers or other students on academic writing.
8. Anything else that is related to your English writing.

Please send your weekly journal within 2 days after each class to the researcher through WeChat or email. Thank you for your cooperation!
